# Evolution of RLSB, a nuclear-encoded S1 domain RNA binding protein associated with post-transcriptional regulation of plastid-encoded *rbc*L mRNA in vascular plants

**DOI:** 10.1186/s12862-016-0713-1

**Published:** 2016-06-29

**Authors:** Pradeep Yerramsetty, Matt Stata, Rebecca Siford, Tammy L. Sage, Rowan F. Sage, Gane Ka-Shu Wong, Victor A. Albert, James O. Berry

**Affiliations:** Department of Biological Sciences, University at Buffalo, Buffalo, NY 14260 USA; Department of Ecology and Evolutionary Biology, University of Toronto, Toronto, Ontario M5S3B2 Canada; Department of Biological Sciences, University of Alberta, Edmonton, AB T6G 2E9 Canada; Department of Medicine, University of Alberta, Edmonton, AB T6G 2E1 Canada; BGI-Shenzhen, Beishan Industrial Zone, Yantian District, Shenzhen 518083 China

**Keywords:** Photosynthesis, S1 domain RNA binding protein, Rubisco *rbc*L gene expression, Land plant evolution, Microsynteny, Whole genome duplication, Gene loss, Single copy gene, Duplication in grasses

## Abstract

**Background:**

RLSB, an S-1 domain RNA binding protein of *Arabidopsis*, selectively binds *rbc*L mRNA and co-localizes with Ribulose-1,5-bisphosphate carboxylase/oxygenase (Rubisco) within chloroplasts of C_3_ and C_4_ plants. Previous studies using both *Arabidopsis* (C_3_) and maize (C_4_) suggest RLSB homologs are post-transcriptional regulators of plastid-encoded *rbc*L mRNA. While RLSB accumulates in all *Arabidopsis* leaf chlorenchyma cells, in C_4_ leaves RLSB-like proteins accumulate only within Rubisco-containing bundle sheath chloroplasts of Kranz-type species, and only within central compartment chloroplasts in the single cell C_4_ plant *Bienertia*. Our recent evidence implicates this mRNA binding protein as a primary determinant of *rbc*L expression, cellular localization/compartmentalization, and photosynthetic function in all multicellular green plants. This study addresses the hypothesis that RLSB is a highly conserved Rubisco regulatory factor that occurs in the chloroplasts all higher plants.

**Results:**

Phylogenetic analysis has identified *RLSB* orthologs and paralogs in all major plant groups, from ancient liverworts to recent angiosperms. *RLSB* homologs were also identified in algae of the division *Charophyta*, a lineage closely related to land plants. *RLSB*-like sequences were not identified in any other algae, suggesting that it may be specific to the evolutionary line leading to land plants. The RLSB family occurs in single copy across most angiosperms, although a few species with two copies were identified, seemingly randomly distributed throughout the various taxa, although perhaps correlating in some cases with known ancient whole genome duplications. Monocots of the order Poales (Poaceae and Cyperaceae) were found to contain two copies, designated here as *RLSB-*a and *RLSB-b*, with only *RLSB-a* implicated in the regulation of *rbc*L across the maize developmental gradient. Analysis of microsynteny in angiosperms revealed high levels of conservation across eudicot species and for both paralogs in grasses, highlighting the possible importance of maintaining this gene and its surrounding genomic regions.

**Conclusions:**

Findings presented here indicate that the RLSB family originated as a unique gene in land plant evolution, perhaps in the common ancestor of charophytes and higher plants. Purifying selection has maintained this as a highly conserved single- or two-copy gene across most extant species, with several conserved gene duplications. Together with previous findings, this study suggests that RLSB has been sustained as an important regulatory protein throughout the course of land plant evolution. While only *RLSB-a* has been directly implicated in *rbc*L regulation in maize, *RLSB-b* could have an overlapping function in the co-regulation of *rbc*L, or may have diverged as a regulator of one or more other plastid-encoded mRNAs. This analysis confirms that RLSB is an important and unique photosynthetic regulatory protein that has been continuously expressed in land plants as they emerged and diversified from their ancient common ancestor.

**Electronic supplementary material:**

The online version of this article (doi:10.1186/s12862-016-0713-1) contains supplementary material, which is available to authorized users.

## Background

As photosynthetic organelles, chloroplasts perform several functions that are ultimately essential for all life on earth. In higher plants and eukaryotic algae, their most biologically significant activities are the conversion of solar energy into organic energy and the release of oxygen. The resulting energy molecules ATP and NADPH support biological carbon fixation, initiated through the carboxylation activity of the chloroplastic enzyme ribulose-1,5-bisphosphate carboxylase/oxygenase (Rubisco) and mediated through the Calvin-Benson cycle [1, 2]. The plastids of higher plants originated from ancient photosynthetic prokaryotes through endosymbiosis approximately 1.5 billion years ago. Organelle evolution has incorporated significant plastid genome reduction, so that only about 100–200 genes are encoded on a small circular genome of approximately 150 kilobases in size. The rest of the 2000–3000 proteins utilized within each chloroplast are encoded by the nuclear genome, translated in the cytoplasm, and imported into the chloroplasts via a plastid targeting/transit sequence [1–3]. Anterograde (nucleus to organelle) and retrograde (organelle to nucleus) signaling processes ensure the coordination of gene expression between the two compartmentalized genomes, so that the protein composition and biological processes confined within the chloroplasts themselves are appropriately integrated with the many other processes occurring throughout the entire plant cell [1].

Plastid-encoded genes are regulated primarily at post-transcriptional levels, with mRNA translation, processing, and stability being primary regulatory determinants [1, 4–7]. Anterograde signaling is dependent on nuclear-encoded, plastid-targeted RNA-binding proteins that interact directly with *cis*-acting sequences of plastid-encoded mRNAs, usually within their untranslated regions (UTRs). There are several classes of sequence-specific binding proteins, the most predominant being the pentatricopeptide repeat (PPR) proteins, with about 450 transcript-specific forms enabling many aspects of RNA metabolism [1, 4]. There are many other types of nuclear-encoded RNA binding proteins that affect chloroplast gene expression, including the CRM, PORR, APO1 families [1, 4], which for the most part have not been well characterized. Recently, the list of categories for RNA binding proteins with demonstrated effects on plastid gene expression was expanded through the identification of the RLSB (***r****bc***L** RNA **S**1-**B**inding domain) protein family, which is defined by its distinct nucleic acid binding domain [8]. RLSB homologs have been associated with post-transcriptional expression of the plastid-encoded Rubisco *rbc*L gene in both C_3_ and C_4_ plant species [8, 9].

Rubisco is the principle enzyme of photosynthetic carbon fixation and is central to the viability, growth, and productivity of all plants. Compartmentalized within chloroplasts, it consists of eight large (LSU, 51–58 kDa) and eight small (SSU; 12–18 kDa) subunits [1, 10, 11]. The LSU-encoding *rbc*L gene is transcribed and translated within chloroplasts, while the nuclear SSU-encoding *Rbc*S gene family is translated on cytoplasmic ribosomes as a precursor containing an N-terminal plastid transit sequence. The *rbc*L and *Rbc*S mRNAs, as well as their encoded proteins, are coordinately regulated so that equal amounts of both subunits accumulate in each chloroplast. Regulation of Rubisco gene expression at post-transcriptional levels, including regulation of mRNA processing (degradation, stabilization, or maturation of transcripts) and control of translation, has been documented in many plant species [1, 10–12]. Post-transcriptional control has been implicated in the regulation and coordination of *Rbc*S and *rbc*L genes in response to a variety of developmental and environmental signals [1, 10, 11]. Post-transcriptional regulation also plays a significant role in the cell-type specific compartmentalization of *rbc*L gene expression in plants that use the highly efficient C_4_ photosynthetic pathway for carbon fixation [1, 10, 11, 13]. This pathway requires that *rbc*L and *Rbc*S gene expression becomes specifically localized to internalized leaf bundle sheath (B) cells that surround the vascular tissue, while the outer layer of photosynthetic cells, the leaf mesophyll (M) cells, do not express either subunit. While multiple examples of post-transcriptional Rubisco regulation have been described, very little is known about specific trans-acting factors involved in the regulation of either subunit. RLSB proteins represent rare examples of potential anterograde regulatory factors associated with post-transcriptional *rbc*L gene expression.

Encoded by the nuclear *RLSB* gene family, RLSB-like proteins appear to be highly conserved among plant species. The S1 binding domain that distinguishes this protein family was first identified in ribosomal protein S1, and is found in a large number of RNA binding proteins [14]. While non-ribosomal proteins known to possess S1 binding domains are widespread among a variety of organisms, including plants, animals, and prokaryotes [8, 14], very little is currently known about the function of most proteins containing this domain. Previous studies identified RLSB orthologs in more than 100 plant species, including eudicots, monocots, C_3_ and C_4_ species; similarities range from 60 % (maize-*Arabidopsis*) to 90 % (maize-sorghum) [8, 9]. All of these contain a plastid transit sequence, and RLSB homologs have been shown to co-localize with the LSU to leaf chloroplasts in both C_3_ and C_4_ plants [8, 9]. Most significantly, RLSB accumulates only within Rubisco-containing chloroplasts of B cells (and not M cells which lack Rubisco) in the leaves of several C_4_ plants, providing additional correlative evidence for its association with *rbc*L gene expression. Even within the unique single-cell C_4_ chlorenchyma cells of *Bienertia sinuspersici* leaves, the RLSB homolog is highly specific to LSU-containing central compartment chloroplasts, and not to peripheral compartment chloroplasts that lack Rubisco [9]. The co-localization of RLSB proteins with Rubisco within the chloroplasts of C_3_ and C_4_ plants, their selective in vitro and in vivo and binding to *rbc*L mRNA, correlation with reduced *rbc*L mRNA and protein accumulation in C_3_ and C_4_ RLSB mutants (as seen in insertion and RNA-silenced lines), and strong conservation across many plant species, provide support for a model in which RLSB proteins function as trans-acting regulatory determinants for Rubisco gene expression in all higher plants [8, 9].

As a step toward understanding how RLSB proteins relate to chloroplast development, *rbc*L gene expression, and overall photosynthetic function within the many different groups of plants, the evolutionary analyses presented here have focused on the distribution, copy number, and variation for genes encoding this protein across a highly diverse sampling of higher plant species. These analyses address the hypothesis that, as a central regulator of Rubisco expression, one or more copies of RLSB-like genes will be present, expressed, and highly conserved across a very broad range of plant genomes. Our findings show that nuclear-encoded *RLSB*-like genes are very highly conserved in higher plants. They occur as a single-copy gene in nearly all of the eudicot species examined, with a few rare species possessing two paralogs seemingly randomly distributed throughout this clade, but possibly correlating with some known ancient whole genome duplication events [15]. Duplications of *RLSB*-like genes were also found in a few lower plant species. However, monocots species in the family Poaceae (grasses) contain a conserved paralog, the function of which has not yet been determined. With regard to the paralogs found in Poaceae, we have designated the previously identified gene associated with Rubisco regulation [8] that is present in all higher plants as *RLSB-a*, and the newly identified grass-specific paralog as *RLSB-b. RLSB-a* was found to occur within a region with high levels of local synteny, suggesting purifying selection has influenced its copy number and regional localization following whole genome duplication events. Our data identify RLSB-like proteins as highly conserved regulatory determinants associated with photosynthetic carbon fixation in all plants, including C_3_ as well as C_4_ species. Understanding RLSB protein family evolution throughout the plant kingdom provides a new window into the evolution of regulatory mechanisms responsible for the synthesis of Rubisco, and accordingly, primary productivity throughout Earth’s biosphere. Identification of molecular evolutionary processes responsible for photosynthetic carbon assimilation is an important step for bioengineering crop plants to enhance food and biofuel production.

## Methods

### Identification and analysis of expressed RLSB orthologs and paralogs in transcriptome databases

Conserved RLSB gene family sequences from a highly diverse range of plant species were obtained using the BLAST [16, 17] tblastn algorithm with *Zea mays* (GRMZM2G087628) and *Arabidopsis* (JX843767) RLSB [8] as query sequences. Multiple databases were screened, including the National Center for Biotechnology Information (NCBI) (http://www.ncbi.nlm.nih.gov), Phytozome 10.2 (http://phytozome.jgi.doe.gov/pz/portal.html#!search), the 1000 Plants project (https://sites.google.com/a/ualberta.ca/onekp/home) and the CoGe server (https://genomevolution.org/coge/). *RLSB*-like orthologs and paralogs were identified as having significant E-values (usually less than 10^−5^) and preserving the known conserved S1 RNA binding motifs. We identified homologs in more than 245 plant species using the aforementioned databases. This search also identified an *RLSB* paralog (*RLSB-b*) in several C_3_ and C_4_ grasses and sedges. Among these sequences, some represented complete full-length mRNAs, while others were scaffolds of partially sequenced genes aligned using the T-coffee sequence (TCS) aligner software (http://tcoffee.crg.cat/apps/tcoffee/index.html). This software revealed conserved regions of the orthologs, particularly within the S-1RNA binding domain region. TCS analysis of the alignment [18] showed a score higher than 85 for all species, indicating a highly reliable alignment.

### Phylogenetic analysis of RLSB gene family transcripts

Orthologous transcriptome sequences were identified in the 1KP datasets and Phytozome 10.2, using BLAST for initial identification and then for validation via reciprocal BLAST back to the *Arabidopsis* sequence. Since *de novo* transcriptome assemblies are often fragmented, when multiple sequences were recovered from a given species the alignment and a preliminary approximate maximum-likelihood tree generated using FastTree were used to manually distinguish fragments from paralogs. If multiple sequences from a single species did not overlap in the alignment and the tree showed no evidence of duplication in that lineage the sequences were assumed to be transcript fragments and were combined in the alignment. If these sequences did overlap, they were assumed to be paralogs if they differed at the amino acid level. Overlapping sequences without amino acid substitutions were assumed to represent allelic variation and were combined. Some key taxa lacked a hit against *RLSB*, e.g., the two hornwort species *Nothoceros aenigmaticus* and *N. vincentianus*, as well as *Welwitschia mirabilis*. For these species, targeted assemblies were attempted using BLAST to identify short reads that mapped to RLSB family protein sequences from related taxa and *de novo* assembly of the identified reads using Geneious. This approach successfully assembled an *RLSB*-like coding sequence for *Welwitschia*, but failed to assemble sequences for the hornworts. It is possible that the hornworts lack RLSB homologs, or that they simply do not express it in tissues that were sampled for RNA-seq.

Bayesian phylogenetic reconstruction was performed on the trimmed amino acid alignment using MrBayes 3.2.5 with 2 independent runs, 32 chains per run. The amino acid substitution model was set to mixed, and thus determined by the MCMC run, and the favoured model was Jones et al. [19]. The analysis was run for over 6.5 million generations, and plateaued with average standard deviation of split frequencies of approximately 4.2 % for the final 2 million generations. Six thousand five hundred trees from these last 2 million generations were sampled from each run and a majority rule consensus tree was made using the consensus program included with ExaBayes 1.4.2, since MrBayes lacks such a stand-alone program. A consensus support threshold of 50 % posterior probability was selected, with nodes below this level of support collapsed.

### Analysis of synteny

Synteny for genomic *RLSB*-like genes and their surrounding regions among major angiosperms species was assessed and visualized using the CoGe server (https://genomevolution.org/CoGe/) as previously described [20].

### Maize lines and growth conditions

For cloning of the maize *RLSB*-*a* and *RLSB*-*b* paralogs, and for mRNA analysis, seeds of wild type B73 and *rlsb-a1/rlsb-a2* Mu-insertion mutant plants (these were previously designated as *rlsb-1/rlsb-2*) were germinated and grown in a growth chamber as described previously [8].

### cDNA cloning and qRT-PCR of maize RLSB paralogs in wild type and mutant maize leaves

To confirm the specificity of primers that were used to independently quantify the accumulation of transcripts encoded from the two maize *RLSB*-like paralogs, total RNA was harvested from the leaves of both wild type B73 and *rlsb-a1/rlsb-a2* Mu-insertion mutant plants, as described previously [8]. cDNA was prepared from these RNA samples using the iScript cDNA synthesis kit (Bio-Rad®) cDNA from each ortholog (designated as RLSB-*a* and RLSB-*b*) was amplified by PCR using primers specific for each transcript. The amplified PCR fragments were then cloned into pDrive vector and transformed into bacteria using a PCR cloning kit (Qiagen®, Hilden, Germany) according to manufacturer’s instructions, for further analysis. PCR amplifications from the cloned fragments were performed in 25 μl volumes with the AmpliTaq DNA Polymerase buffer II kit (Applied Biosystems, Foster City, CA, USA) using 2.5 μl buffer, 2.5 μl MgCl_2_, 1.0 μl dNTP, and 0.6 μl each of M13F and M13R primers and 0.2 μl of AmpliTaq polymerase. All PCR products were examined by gel electrophoresis on 1 % agarose gels, and the insert-containing PCR-positive plasmids were sequenced in one direction using M13 primer at the Roswell Park Cancer Institute sequencing facility (http://biopolymer.roswellpark.org). The sequencing results from several independent clones were analyzed using BLAST to confirm that they corresponded to one or both of the maize RLSB paralogs.

### Analysis of *RLSB*-*a* and *RLSB*-*b* transcript accumulation by qRT-PCR

As described previously, leaf 3 (these were referred to as second emerging leaves in [8]) from wild type B73 and Mu-insertion mutant maize plants were harvested at 12–13 inches in length. These were divided into 7 equidistant sections (from the base of the leaf to the tip) for analysis of RLSB-*a* and RLSB-*b* mRNAs in the different leaf sections. Total RNA was harvested from each section, according to methods previously described [8]. cDNA was prepared from these RNA samples using iScript cDNA synthesis kit (Bio-Rad) with primers specific for *RLSB*-*a* (left primer, CCACTTCCATAACCCAGCAT and right primer, ATTTCACTCCAGGGGCACTA) and *RLSB*-*b* (left primer, ATCAACAGAAGAAGCGCTCG, and right primer, TAACTAACCCCACGCTCACC). Levels of mRNA for *RLSB-a* and *RLSB-b* in the different leaf sections was determined using qRT-PCR, and standardized to actin mRNA. Quantification of transcript levels in both cases was calculated using ΔΔCt method, standardized to actin mRNA. Data was averaged for three wild type and three mutant siblings, with three technical repeats for each of the plant samples. The differences in expression levels of *RLSB-a* and *RLSB-b* in each of the seven leaf sections of both wild type and mutant plants, from all the repeats was subject Student’s *t*-test to ensure the *P* values were lower than 0.05, which denotes the statistical significance of the data. Correlation analyses done separately for the expression level data from all the section denoting yellow bases and from the sections denoting the green tip regions of the mutants maize leaves yielded similar results with r^2^ values of 0.002 and 0.338 respectively, suggesting absence of significant correlation between the expression levels of *RLSB-a* and *RLSB-b* in mutants plants.

## Results

### RLSB family gene transcripts are present and conserved in all vascular plant groups

*Arabidopsis* and maize *RLSB* cDNA sequences [8] were used in a comparative search for expressed orthologs in other angiosperm species using the BLAST tblastn algorithm as a search tool with several plant transcriptome databases. Transcripts were identified as being encoded by RLSB orthologs based on having significant E values (less than 10^−5^) and by containing alignable sequence outside the S1 binding motif. The data were compiled mostly from available complete transcriptomes. Data from some plant groups, such as algae, bryophytes, marchantiophyta, lycophytes, ptreridophytes, gymnosperms and angiosperms, were derived from partial transcriptomes or complete transcriptomes based on their availability.

**Fig. 1:**
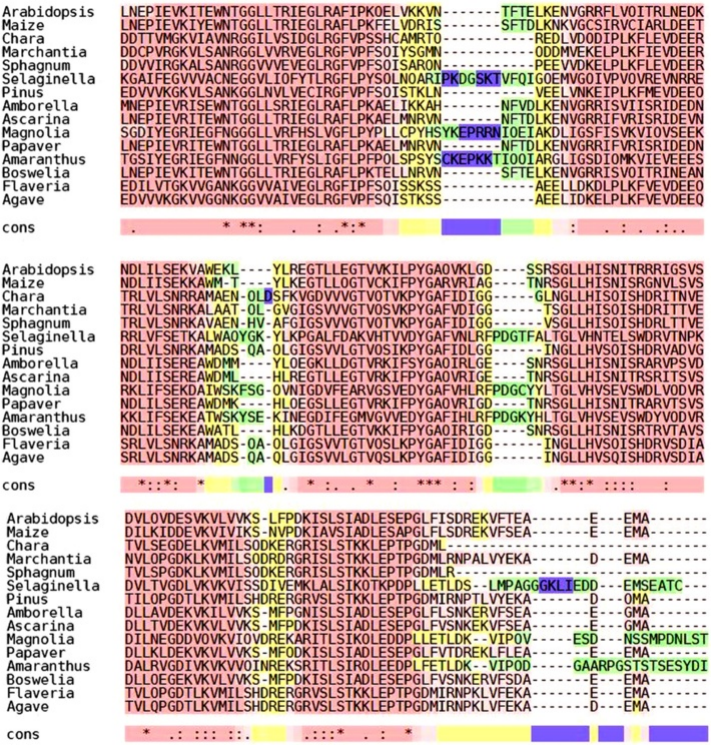
*RLSB* homologs are present and conserved across a broad range of plants species. *Arabidopsis* and maize RLSB family transcripts were used as reference sequences to search for orthologs in a broad assortment of plant species representing major plant groups, ranging from algae to angiosperms. Representative species included in the alignment shown here are *Chara vulgaris* and *Saragassum thunbergi* representing algae, *Marchantia palaeceae*, *Sphagnum recurvum* representing liverworts and mosses respectively, *Sellaginella sellaginoides*, a lycophyte, *Pinus ponderosa*, a gymnosperm, *Amborella trichopoda,* representing basal angiosperms, *Ascarina rubricaulis*, belonging to the order *Chloranthales* of Angiosperms, *Magnolia grandiflora*, representing magnolids, *Papaver somniferum*, an early eudicot, *Boswellia sacra* and *Flaveria bidentis* represent the core eudicots, and *Agave tequiliana* is a monocot that also utilizes CAM photosynthesis. The BLAST search using the tblastn algorithm in the 1000 Plants database (https://www.bioinfodata.org/Blast4OneKP/blast) revealed scaffolds in various plant species with sequence similarity to RLSB family members. Multiple sequence alignment using T-coffee sequence aligner software (http://tcoffee.crg.cat/apps/tcoffee/index.html) shows the most conserved regions within the homologs, including the S-1 binding domain region. TCS analysis of the alignment [18] showed a score higher than 85 for all species, indicating a highly reliable alignment. Note that less conserved regions at the N-terminal and C-terminal areas are not shown in this figure

RLSB gene family homologs were found in nearly all of the lineages analyzed examined between the land plants and Klebsormidiales, with the exception of Anthocerophyta (hornworts) and coleochaetales. Given that RLSB proteins are highly conserved, and the fact that knockout mutants of higher plants have severe photosynthetic deficiencies, this absence is likely due to lack of expression in the tissues sampled for transcriptome sequencing. This included all of the major plant groups, including green algae (charophytes), non-vascular plants (bryophytes), pteridophytes, gymnosperms, and angiosperms (https://www.bioinfodata.org/Blast4OneKP/blast). Figure 1 shows the *Arabidopsis* and maize sequences aligned with several plant species representing the major plant groups, from algae to angiosperms. Representative species included in this alignment are *Chara vulgaris* representing green algae, *Marchantia palaeceae* and *Sphagnum recurvum* representing bryophytes, *Sellaginella sellaginoides*, a lycophyte, *Pinus ponderosa*, a gymnosperm, *Amborella trichopoda,* representing basal angiosperms [21], *Ascarina rubricaulis*, belonging to the order Chloranthales of Angiosperms, *Magnolia grandiflora*, representing magnolids, *Papaver somniferum,* a basal eudicot [22], *Boswellia sacra* and *Flaveria bidentis* representing the core eudicots, and *Agave tequiliana*, a monocot that also utilizes CAM photosynthesis [23].

**Fig. 2:**
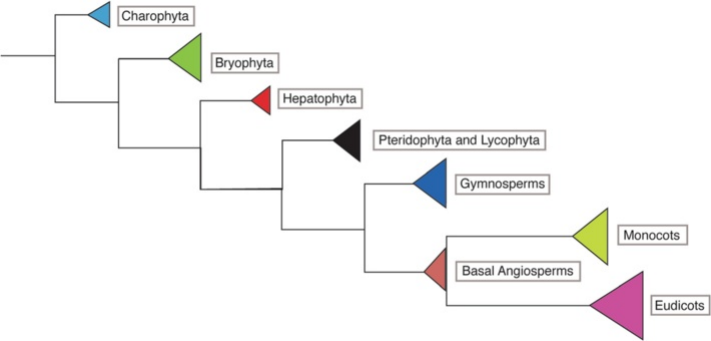
A condensed cladogram representing all the major groups of plants in which RLSB homologs have been identified. The phylogeny shows the major land plant groups in which RLSB gene family members were found. The *Arabidopsis RLSB* transcript was used as reference sequence in BLAST search for orthologs across all groups of plants. Sequences from different plant species were obtained and were aligned with the *Arabidopsis* RLSB protein. The aligned amino acid sequences were used to generate a phylogram rooted by Charophyta as described in materials and method

**Fig. 3:**
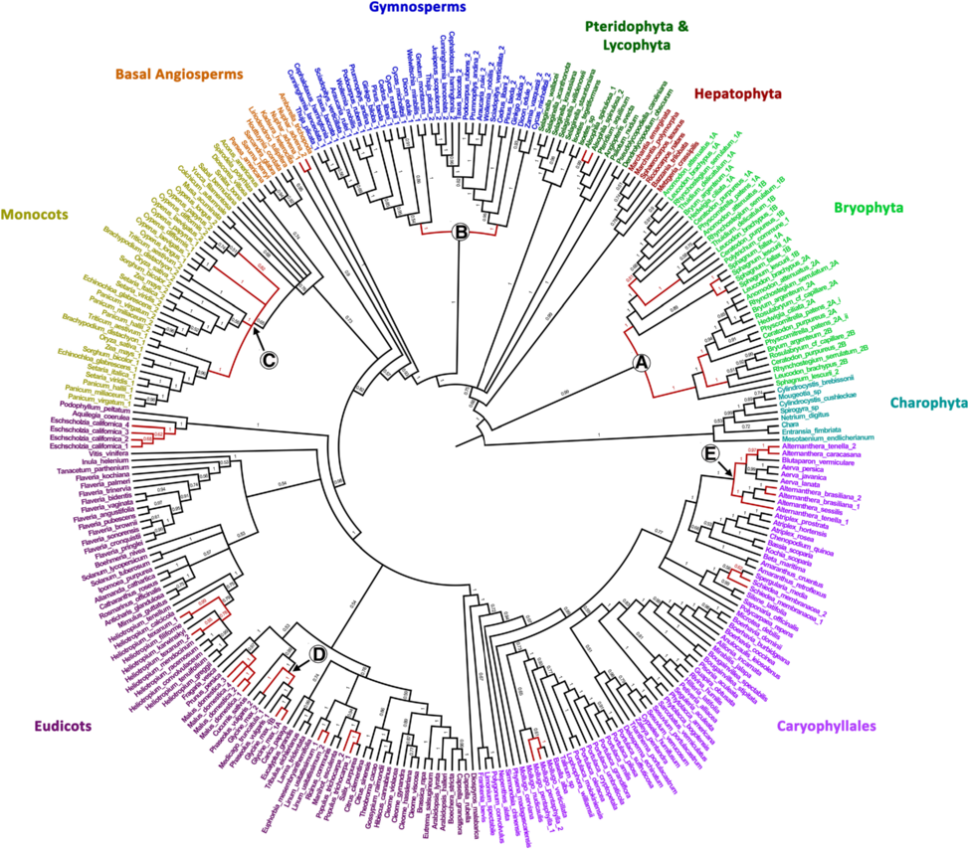
Bayesian gene tree showing the presence of transcripts encoded from RLSB homologs in land plants and Charophyte algae. Branch labels are posterior probabilities. Branches representing gene duplication or leading to duplicate copies are highlighted in *red*. Major events include: *A*, basal duplication in all Bryophytes, followed by subsequent duplication in Bryopsida and Sphagnum; *B*, basal duplication in all extant gymnosperms (although orthologs were not found in the RLSB2 gymnosperm clade for Gnetales); *C*, duplication in both the grass (Poaceae) and sedge (Cyperaceae) families (lack of support makes it unclear whether this represents duplication in each family or a single duplication event in a shared ancestor); *D*, duplication in Fabaceae; and numerous smaller-scale duplication events; E, the presence of two distinct and well-supported Alternanthera clades which are not sister to one another suggests duplication and loss within Amaranthaceae

The condensed cladogram shown in Fig. 2 identifies the major groups of plants in which RLSB homologs have been found. A more comprehensive phylogenetic tree showing each of the individual species examined and relative branch strength values is shown in Fig. 3. From these data, it is evident that *RLSB*-like genes are present in a large number of species across all major plant groups of land plants. Extending our previous findings [8, 9], angiosperm species expressing RLSB family genes included both monocots (such as rice, maize, *Setaria*, *Brachypodium* etc.)*,* and eudicots (such as *Arabidopsis Amaranthus*, *Flaveria*), and in both C_3_ and C_4_ plants.

In addition to the higher plants, *RLSB*-like sequences were identified in several lower plant species, including mosses, liverworts, bryophytes, lycophytes, and ferns (Figs. 2 and 3). *RLSB*-like transcript sequences were identified in eight Charophyte algae, from the Zygnematophyceae (recognized as the closest extant lineage to the land plants, [24–27]), Charales, and Klebsormidiales (the most distant relative of the Embryophyta in which an ortholog was found) (Figs. 1 and 3). Sequences were not identified in any other algae species examined, including aquatic Chlorophyte (*Chlamydomonas reinhardtii)* and brown (*Saragassum thunbergi*) algae. The finding that RLSB homologs are present in Charophyte lineages, considered to be closely related to the common ancestor of all vascular plants [24–27], as well as all other non-vascular plant groups examined, indicates an ancient origin for this conserved protein family that appears to have preceded the invasion of terrestrial environments.

All of the complete full-length *RLSB*-like gene sequences were found to encode a chloroplast transit sequence, indicating that their gene products are targeted to chloroplasts (Additional file 1: Figure S1). It should be noted that orthologs that did not indicate a transit peptide were derived from species with incomplete sequence assemblies (such as *C. vulgaris*). The widespread presence of conserved, plastid-targeted *RLSB*-like sequences in all of these major plant groups, including charophyte algae, strongly supports a significant and conserved regulatory function for this gene family within the chloroplasts of all plants. These findings are consistent with previous immunolocalization and cell-separation studies showing that RLSB protein homologs are localized to Rubisco-containing chloroplasts in several C_3_ and C_4_ dicot and monocot species [8, 9]. Taken together with studies demonstrating the association of RLSB-like proteins within the C_3_ dicot *Arabidopsis* and the C_4_ monocot maize [8], findings presented here provide evidence that the family has maintained an essential and conserved role in chloroplast function and the regulation of *rbc*L expression throughout plant evolution.

To search for the occurrence of potential ancestral S1 domain regulatory proteins in organisms that significantly predate the *Charophyte*-based monophyletic lineage leading to higher plants, a BLAST search of several representative prokaryotic organisms was conducted using the *Arabidopsis RLSB* transcript as a reference (see Additional file 2: Table S1). Stringent search parameters (E = less than 10^−5^) identified protein sequences with very low sequence similarity to the S1-RNA binding domain itself in some purple non-sulfur bacteria (*Rhodospirillum*, *Rhodopseudomonas*, etc.), a class of phototrophic bacteria that perform photosynthetic carbon assimilation through Rubisco-like proteins consisting of only LSU subunits [28]. Since these similarities occur only near the C-terminal portion of the proteins that contains the S1-binding domain, the relatedness to RLSB-like proteins is limited only to their basic nucleic acid binding function. Regulatory functions of prokaryotic S1-RNA binding have been shown to play a role in the expression of several essential bacterial genes by binding to the 5′end of their mRNA to modulate translational initiation or elongation [29–31]. However, an effect on LSU expression for these proteins has not been investigated in these photosynthetic prokaryotes. While it is evident that S1-RNA binding domains do play an important role in prokaryotic as well as eukaryotic gene regulation [8, 14, 31], the current data cannot establish a direct evolutionary relationship between these prokaryotic proteins and the chloroplastic RLSB homologs present in eukaryotic plants.

### RLSB gene family transcripts are present as a single copy in most eudicots, and in two copies in many monocot grasses and sedges

Using the *Arabidopsis RLSB* sequence as a reference, a BLAST search identified RLSB gene family transcripts in all angiosperm species for which data were available. This included *RLSB*-like sequences in the early-diverging angiosperm species *Amborella*, to more recent lineages within eudicots and monocots (Figs. 1, 2 and 3). Nearly all of the eudicots included in this analysis had a single copy of the RLSB gene family. However, there were a few rare species among the eudicots that were found to possess two copies (Fig. 3). Examples were the Fabaceae (*Glycine max*), Phrymaceae (*Mimulus*), and Arecaceae (*Phoenix dactylus*). These were seemingly randomly distributed among families, with no clear taxonomic correlation, although they might be related to some known ancient whole genome duplications [15]. An independent RLSB family duplication was also found at the base of gymnosperms, including taxa such as *Ginkgo biloba*, *Cedrus libani, Cycas micholitzii, Cunnighamia lanceolata*, *Pinus taeda, Cedrus libani*, *Taxus baccata*. Similar deep duplications were also visible in bryophytes such as *Rynchostegium serrulatum*, *Physcomitrella patens, Sphagnum lescurii, Bryum argenteum,* and *Ceratodon purpureus.* In angiosperms, the only clear lineage-wide duplication appeared among monocot species within the family *Poaceae* (grasses) and *Cyperaceae* (sedges), where a conserved paralog is retained whose function has not yet been determined (Fig. 3). In rice and maize the translated protein sequence similarities between these paralogs ranged from approximately 50 to 60 %, respectively. The change from one *RLSB*-like gene to two paralogs in these commelinid monocots was most likely related to a whole genome duplication (WGD) event that occurred during evolution of the lineage around 70 to 100 million years ago (MYA) [32, 33], followed by chromosomal rearrangements and fusions [34]. Most of the basal monocot species, such as *Spirodela* and *Musa*, show only one copy from RLSB gene family. Regardless of their duplication status, conservation of *RLSB* homologs was very high across the range of angiosperm species examined. These findings demonstrate that the RLSB gene family has been strictly maintained, at the very least for a single-copy, canonical function. Indeed, this high degree of conservation not only among angiosperms but across all land plants suggests strong purifying selection acting since their early evolution as well as through their subsequent radiations.

### *RLSB* homologs share microsynteny in several angiosperm species

**Fig. 4:**
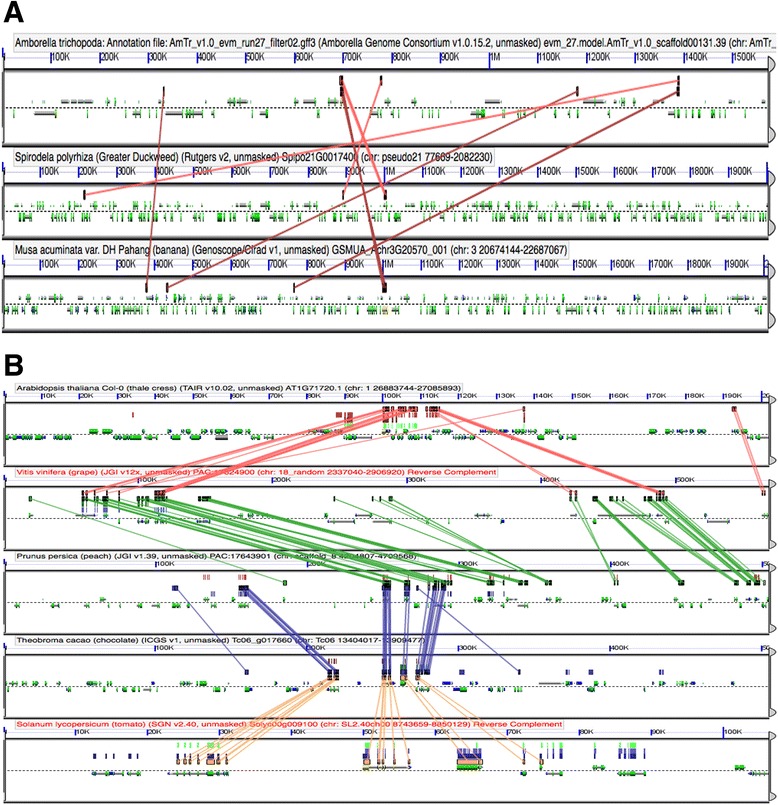
Synteny between basal angiosperms, monocots and eudicots. Panel **a** shows microsynteny for a region spanning 1.5 Mb in *Amborella* and 1.9 Mb in the representative monocots *Spirodela* and *Musa*, around the RLSB gene locus. Panel **b** reveals strong microsynteny between regions around RLSB in the representative eudicots *Arabidopsis thalliana*, *Vitis vinifera*, *and Prunus persica*, *Theobroma cacao*, and *Solanum lycopersicum* (1.9 Mb). Any regions of similarity present are denoted by colored boxes above the genomic area of similarity and these are connected by colored lines between the genomes of different species

**Fig. 5:**
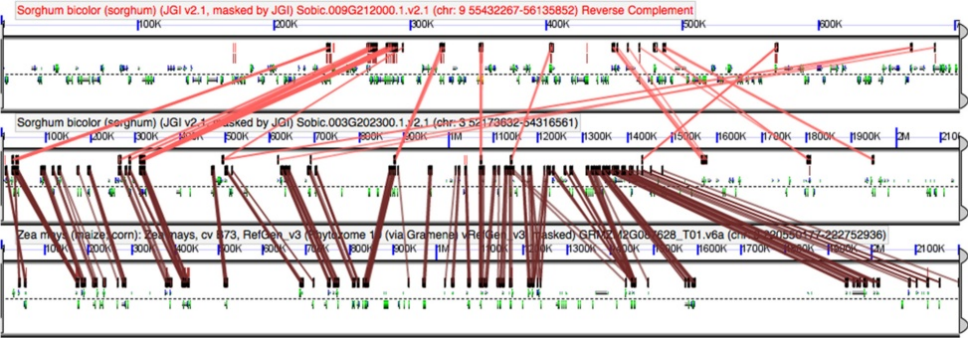
Conserved microsynteny between the two paralogs in representative grass species. High levels of synteny occur within the regions spanning ~1 Mb on either side of RLSB paralogs (*RLSB-a* and *RLSB-b*) in grasses, represented in this figure by *Sorghum bicolor*, which were also compared to *RLSB-a* of *Zea mays* as a reference. A large amount of synteny is indicted by the presence of numerous colored boxes in the stretch of genome surrounding the two paralogs and these matching regions being paired with each other by the connecting colored lines

In many of the plant species examined, conservation of RLSB gene family sequences was accompanied by conservation of the local genome structural region (microsynteny). Analysis of synteny was performed using CoGe (https://genomevolution.org/CoGe/) (Figs. 4a, b, and 5). The single *RLSB* homolog in the early diverging angiosperm species *Amborella* showed only weak microsynteny with corresponding blocks in monocots (Figs. 4a), while eudicot genomes showed no detectable synteny with the same *Amborella* region. It is likely that the relatively weak synteny observed between *Amborella* and monocots is representative of considerable gene loss and/or rearrangements occurring over deep evolutionary time [35–37]. Nonetheless, all the eudicot species examined showed relatively strong internal synteny when compared to each other (Fig. 4b), suggesting that the genomic block containing the *RLSB* ancestor has been well conserved structurally (and possibly selectively) within this major group of higher plants. For the monocots, it is interesting to note that in the grass lineage a very high level of internal synteny exists between its two paralogs, even though these genes and surrounding regions are located on different chromosomes in certain species (Chromosome 3 and 6 in maize, Chromosome 3 and 9 in *Sorghum* or different scaffolds in case of *Setaria*) (Fig. 5). This finding of internal synteny for the grass paralogs is consistent with a model in which the two RLSB gene family copies resulted from one of the known WGD events in grasses [32, 33], with both duplicates and their surrounding regions being retained. In some cases, such duplications can lead to neofunctionalization, such that one paralog retains its original function, while the other paralog is free to acquire a new functional role [38, 39]. An alternative possibly for duplicate retention is subfunctionalization, in which necessary ancestral roles are partitioned among duplicate copies retained by purifying selection.

### The two RLSB paralogs in maize

The occurrence of two RLSB paralogs in grasses and some other monocots raises a question about the function of two paralogs in these species. To address this question, the expression of the two maize paralogs *RLSB-a* and *RLSB-b* was examined in leaves from wild type as well as the Mu insertion mutant plants described previously [8]. The qRT-PCR mRNA analysis utilized primer sets carefully chosen to make sure that they would only amplify either *RLSB-a* or *RLSB-b* transcripts, but not both (Additional file 3: Figure S2). The primers were first used to amplify each transcript from wild type maize leaf cDNA. Each of the amplified PCR products were then cloned, and several independent clones were sequenced to confirm specificity of the primer sets for each of the paralogs (Additional file 3: Figure S2).

**Fig. 6:**
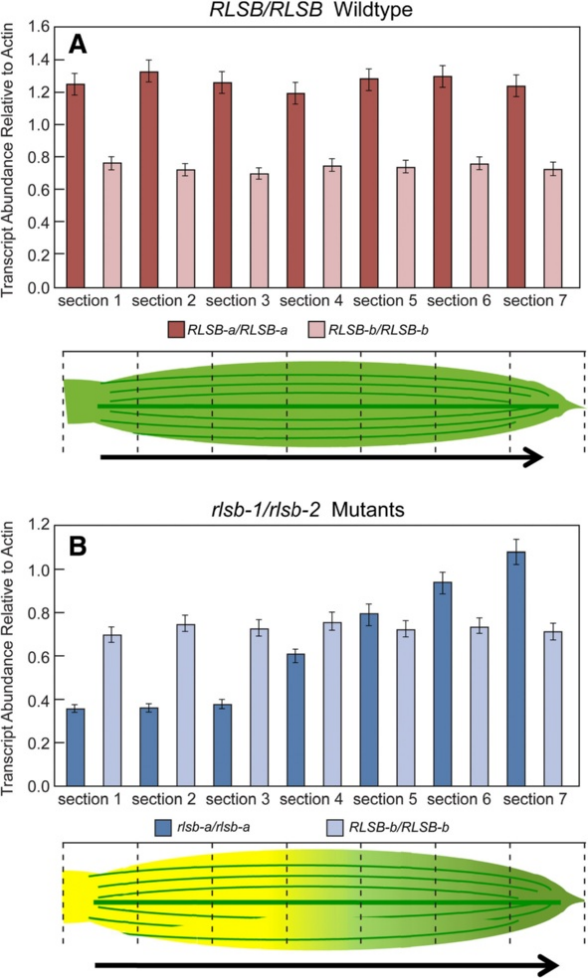
Levels of *RLSB-a* and *RLSB-b* transcript accumulation across maize leaf developmental gradients in wild type and *rlsb-a1/rlsb-a2* insertion mutant plants. The transcription levels of the two maize RLSB paralogs *RLSB-a* and *RLSB-b* across wild type and mutant gradients maize leaf gradients were analyzed by qRT-PCR using primers highly specific for each sequence, as described in Methods. Sections used for sampling along the leaf gradients are indicated by dashed lines. Panel **a** Relative levels of mRNA accumulation for *RLSB-a* and *RLSB-b* transcripts in wild type *RLSB/RLSB* maize seedlings. Panel **b** Relative levels of mRNA accumulation for *RLSB-a* and *RLSB-b* transcripts in *rlsb-a1/rlsb-a2* mutant maize seedlings. Quantification of transcript levels in both cases was standardized to actin mRNA. Data was averaged for three wild type and three mutant siblings, with three technical repeats for each of the plant samples. Statistical significance was calculated using Student’s *t*-test, as described in materials and methods. Note that the expression levels of *RLSB-a* and *RLSB-b* from the seven sections of the mutant plants show very little correlation (*r* ^*2*^ = 0.335), suggesting a markedly different trend in the expression patterns of these two homologs in the mutant plants. For each bar, P values were less than 0.05

Once the primer set specificity was confirmed, qRT-PCR analysis was used to determine the expression profile for each paralog along the progressive developmental gradient of 12–13 cm maize leaves (Fig. 6). Levels of *RLSB-a* and *RLSB-b* mRNA were analyzed within seven sections taken along the length of each leaf. For the orientation of the graphs shown in Fig. 6, section 1 was from the base of the leaf, section 4 was at the mid section, and section 7 was at the apex. In wild type leaves, both paralogs showed only slight variation in their levels of mRNA accumulation across the entire leaf gradient, with *RLSB-a* about one half to one third more abundant than *RLSB-b* in each section (Fig. 6a). In leaves of the *rlsb-a1/rlsb-a2* mutant, which shows reduced expression due to the Mu insertions within each copy of the *RLSB-a* gene [8], levels of *RLSB-a* mRNA were lower at the leaf base, and increased along the length of the leaf to about a three fold increase at the apex. This is in agreement with previous findings showing that maize *RLSB* mRNAs are less abundant at the base and more abundant at the apex of leaves in the insertion mutants [8]. In contrast, transcripts encoded by the *RLSB-b* paralog (which does not contain a Mu insert) in these same leaf sections, did not show any variation along the gradient, with levels similar to that of wild type plants across the entire length of the mutant maize leaf (Fig. 6b). Thus, at the base of the mutant leaves, *RLSB-a* was less abundant that *RLSB-b,* while at the apex *RLSB-a* had increased to become the more abundant transcript. Changes in *RLSB-a* mRNA levels across the leaf gradient of the mutant leaves correlate with the changes in levels of *rbc*L mRNA and protein that were reported in our earlier study [8]. However, *RLSB-b* transcript levels remained constant in all regions of the mutant leaves, and thus in itself showed no correlation with the reduced *rbc*L expression previously observed at the lower leaf regions. It should be noted that maize leaf 3 used for these analysis is of embryonic origin, and *RLSB-a* and *RLSB-b* expression patterns could differ in later leaves or other photosynthetic tissues. These findings suggest that the two maize paralogs, although highly similar in terms of their sequence and local surrounding genomic environment (microsynteny), might have diverged from each other to acquire different functions in maize and possibly also in other C_4_ grasses.

## Discussion

The nuclear-encoded RLSB gene family produces mRNA binding proteins that are targeted to chloroplasts. Their defining S1 binding site is found in many other nucleic acid binding proteins, including many non-ribosomal proteins as well as some components of the chloroplast ribosomes [8, 14, 40–42]. However, outside of the conserved S1 binding domain, comparative sequence analysis demonstrates that RLSB proteins are very distinct from other known members of the protein superfamily, including ribosomal protein S1 and the recently identified ribosomal protein SDP [40, 41] (Additional file 4: Figure S3). While little is known about the function of most non-ribosomal S1 domain proteins in plants and other organisms, previous studies have linked RLSB homologs with the expression of the plastid-encoded *rbc*L gene in C_3_ and C_4_ plant species [8, 9].

Findings presented here indicate that RLSB homologs are present across the entire range of vascular plants, and are highly conserved even among evolutionarily divergent species. While most plant species possess only a single copy of this gene, gymnosperms, bryophytes, some eudicots and many species of Poaceae possess two conserved paralogs. Analysis of synteny indicates the local genomic region surrounding these genes and their paralogs are also conserved in many species. This analysis provides evidence that RLSB gene family sequence, copy number, and dosage have been strongly conserved throughout the evolution of land plants. Findings from these evolutionary analyses, together with the demonstrated role of RLSB-like proteins in the post-transcriptional regulation of plastid-encoded *rbcL* mRNAs, and the fact that reduced gene expression in both *Arabidopsis* and maize leads to severe photosynthetic impairment or lethality [8], provide compelling evidence that the RLSB family is an essential determinant of chloroplast function, *rbc*L expression, and photosynthesis in all plants.

All major groups of plants, including mosses, ferns, liverworts, gymnosperms and angiosperms, are thought to have originated as a monophyletic group from an ancient charophyte-like green alga between 450–500 million years ago [24–27]. RLSB family sequences were identified in several Charyophytes, but not in other green algae such as *Chlamydomonas reinhardtii* (a single cell green marine algae) or the phaeophyte *Saragassum thunbergi* (multicellular aquatic brown algae) (see Additional file 2: Table S1, for a list of bacterial and algal species examined). It is possible that the ancestral RLSB mRNA binding protein may have become established and maintained as a nuclear-encoded, plastid-targeted protein in an ancestral charophyte species. If this scenario is correct, then the proposed regulatory function of the protein on plastid-encoded *rbc*L mRNA could have originated either in an early charophyte, or possibly at some later point during evolution of the now-extinct stem lineage of land plants.

It is worth noting that S1 binding proteins with potential regulatory capability have been found in several prokaryotes, including *Rhodopsedomonas palustris,* a prokaryotic organism capable of photosynthetic carbon fixation [28–31]. Interestingly, this organism has a Rubisco enzyme that is composed of LSU-like proteins that complex as a homodimer [43]. This would imply that RLSB proteins, which bind *rbc*L mRNAs, could in fact have a very ancient origin that preceded their establishment in photosynthetic eukaryotes. Although true RLSB homologs were not identified in any prokaryotic species examined, including cyanobacteria, these lineages do possess other S1-domain proteins. It is possible that lateral gene transfer from an endosymbiont-derived primordial chloroplast possessing an early S1-domain RNA binding protein could have led to its incorporation/modification as a nuclear-encoded regulatory gene during a very early stage of plant cell evolution, and that one of these S1 proteins subsequently gave rise to the RLSB gene family via duplication and neofunctionalization This mechanism has been proposed for many nuclear-encoded plastid genes, including some involved in chloroplast regulation and translation [1, 4, 44–47].

*RLSB* homologs and their surrounding genomic regions occur in duplicate in maize and many other monocot grasses. This is consistent with an early whole genome duplication that occurred at the base of the order Poaceae [48, 49], followed by the subsequent relocation of the duplicated block in cases such as maize where they exist on different chromosomes in the modern genome [50]. This duplication has been maintained in modern grass species, suggesting that adaptive advantage (through neofunctionalization of one duplicate) or functional partitioning (via subfunctionalization) has led to the two *RLSB*-like paralogs becoming fixed within the genomes of this clade [38, 50–52]. Each of these processes would be consistent with the finding that *RLSB–a* and *RLSB-b* are both expressed without significant variation across the entire maize leaf gradient (Fig. 6), while inactivation of *RLSB-a*, in itself, was sufficient to cause reductions in *rbc*L mRNA and protein accumulation in the maize Mu insertion lines described previously [8]. If the two paralogs have diverged to recognize different binding/regulatory mRNA targets (neo-functionalization), then *RLSB-a* might be specifically associated with *rbc*L transcripts, while *RLSB-b* could be associated with another as-yet unidentified plastidic mRNA. In another form of neofunctionalization, one paralog might have acquired a novel pattern of cell or tissue-specific expression [52]. This could have led to the development of divergent patterns of functionalities, with one of the gene duplicates being more active at the leaf base and the other at the leaf tip. It is also possible that the two paralogs have diverged but are both still required for binding/regulation of *rbc*L mRNA within the same leaf cells (subfunctionalization), perhaps associating together as an RNA binding heterodimer. In this case, the loss of function for one of the two interacting proteins would be enough to cause loss of function for the entire heterodimer, leading to the *rbc*L mRNA and protein reductions observed in the mutant maize lines. If both paralogs have retained their original function (conservation of function), then each of the *RLSB*-like genes might serve identical complementary roles in *rbc*L mRNA metabolism, with both copies required for optimal (maximized) *rbc*L expression in these monocot leaves. This might explain the “leakiness” of the *RLSB-a* mutants, if the residual *RLSB* mRNA and protein were in fact produced only from the non-mutated *RLSB-b* paralog. Distinguishing between these different mechanisms will be resolved by additional functional analysis of both *RLSB-a* and the newly identified *RLSB-b* paralog in wild type and *RLSB-b* mutant maize leaves.

Its high microsynteny within eudicots and between grass subgenomes from an ancestral WGD in surrounding genomic regions, strong sequence conservation, and low copy number distinguish the RLSB gene family from the abundant and diverse PPR class of chloroplast RNA binding proteins. While RLSB-like genes occur only as single or few copies, there are otherwise more than 450 members of the extensive PPR gene family in higher plant genomes [1, 53]. These show many variations in sequence and function, with different members involved in RNA editing, transcript processing, and other RNA metabolic functions. In apparent contrast to the RLSB family, many PPR genes appear to show little or low synteny in their surrounding regions, even for PPR genes relatively closely linked on the same chromosomes [54]. Although we have not characterized this data ourselves, this finding by others could suggest that PPR protein genes may have commonly been subject to diversifying selection, resulting in multiple paralogs and orthologs that vary in function. In contrast, evidence presented here suggests that negative selection has preserved RLSB gene family sequences, limited their functional divergence, and maintained their microsynteny across a very wide range of plant species.

## Conclusions

Nuclear genes encoding the unique plastid-targeted RLSB S1 domain *rbc*L-mRNA binding protein are present and expressed across a wide array of plant species. The highly conserved RLSB gene family appears to have originated very early in the evolution of land plants, possibly in a common ancestor of charophytes and higher plants. *RLSB* homologs have been maintained as a single- or duplicate copies in all land plant species, with conserved duplications of RLSB and its surrounding genomic regions distributed throughout the taxa, most notably in monocot grasses and sedges. Of the two paralogs in Maize, only *RLSB-a* has been directly implicated in *rbc*L regulation. *RLSB-b* could have an overlapping function in the co-regulation of *rbc*L, or may have diverged to function as a regulator of one or more other plastid-encoded mRNAs. Taken together with previous findings (8,9) this study provides strong evidence that RLSB-like genes have been conserved and sustained at low copy number throughout the course of land plant evolution. Evidence presented here provides strong support for the conservation of RLSB as a critical regulator of photosynthetic function as the evolutionary lineages of higher plants emerged and diversified from their ancient common ancestor. This study represents the most thorough evolutionary analysis of any member of the S1 class of nucleic binging protein to date.

## Abbreviations

B, bundle sheath; LSU, rubisco large subunit protein; M, mesophyll; MYA, million years ago; PET, photosynthetic electron transport; PPR, pentatricopeptide repeat; RLSB, *rbc*L RNA S1-binding domain protein; Rubisco, ribulose-1,5-bisphosphate carboxylase/oxygenase; SSU, rubisco small subunit protein; UTR, untranslated region; WGD, whole genome duplication

## Additional files

Additional file 1: Figure S1. CloroP prediction of chloroplast transit sequences. Data shown in in this table are from the ChlorP prediction tool, and predict the presence of chloroplast transit sequences in RLSB orthologs from representative species from different groups of land plants. The presence of a chloroplast transit peptide was revealed in all plants for which complete full length RLSB transcript sequences were available. (PDF 177 kb) Additional file 2: Table S1. List of representative bacterial and algal species examined for the presence of RLSB-like proteins. (PDF 1716 kb) Additional file 3: Figure S2. The primer sets used to amplify regions specific only for *RLSB-a* and for *RLSB-b* are shown. The amplified product sizes are 150 and 153 bp respectively. The regions where the left and right primers bind are indicated by arrows. (PDF 162 kb) Additional file 4: Figure S3. Multiple sequence alignment using T-coffee sequence aligner software (http://tcoffee.crg.cat/apps/tcoffee/index.html) of the *Arabidopsis* SDP S1 ribosomal protein and RLSB shows that these two proteins share only very little sequence similarity, identifying as distinct proteins. (PDF 533 kb)
