# Supplementary material for: Evolution of RLSB, a nuclear-encoded S1 domain RNA binding protein associated with post-transcriptional regulation of plastid-encoded rbcL mRNA in vascular plants
Source: BMC Evol Biol. 2016 Jun 29;16:141. doi: 10.1186/s12862-016-0713-1 (PMC4928308; doi:10.1186/s12862-016-0713-1)
Supplement: Additional file 1: Figure S1. — CloroP prediction of chloroplast transit sequences. Data shown in in this table are from the ChlorP prediction tool, and predict the presence of chloroplast transit sequences in RLSB orthologs from representative species from different groups of land plants. The presence of a chloroplast transit peptide was revealed in all plants for which complete full length RLSB transcript sequences were available. (PDF 177 kb) [file 12862_2016_713_MOESM1_ESM.pdf]

Additional File 1: Figure S1

Name	Length	Score	cTP	CS-score	cTP-length
Arabodopsis	487	0.518	Y	5.063	63
Sphagnum	360	0.527	Y	3.808	16
Ph.patens	414	0.563	Y	1.284	63
selaginella	434	0.577	Y	4.327	64
Populus	528	0.506	Y	-0.785	76
S.bicolor	426	0.554	Y	5.420	45
Kalanchoe	506	0.561	Y	-1.476	69
Musa	417	0.567	Y	5.597	43
