# Supplementary material for: Evolution of RLSB, a nuclear-encoded S1 domain RNA binding protein associated with post-transcriptional regulation of plastid-encoded rbcL mRNA in vascular plants
Source: BMC Evol Biol. 2016 Jun 29;16:141. doi: 10.1186/s12862-016-0713-1 (PMC4928308; doi:10.1186/s12862-016-0713-1)
Supplement: Additional file 2: Table S1. — List of representative bacterial and algal species examined for the presence of RLSB-like proteins. (PDF 1716 kb) [file 12862_2016_713_MOESM2_ESM.pdf]

Additional File 2: Table S1

ALGAE			
**Organism name**	**Division**	**Sample code**	**website link**
Sargassum thunbergii	Phaeophyta	YRMA	https://www.bioinfodata.org/Blast4OneKP/species?id=74
Laminaria japonica	Phaeophyta	QDTV	https://www.bioinfodata.org/Blast4OneKP/species?id=57
Chlorella minutissima	Clorophyta	MWAN	https://www.bioinfodata.org/Blast4OneKP/species?id=742
Chlamydomonas reinhardtii (Chlamydomonas smithii)	Chlorophyta	3055	http://www.uniprot.org/blast/uniprot/B201605198A530B6CA0
Roya obtusa	Clorophyta	XRTZ	https://www.bioinfodata.org/Blast4OneKP/species?id=805
Penium margaritaceum	Chlorophyta	AEKF	https://www.bioinfodata.org/Blast4OneKP/species?id=821
Klebsormidium subtile	Chlorophyta	FQLP	https://www.bioinfodata.org/Blast4OneKP/species?id=791
Mesotaenium endlicherianum	Chlorophyta	WDCW	https://www.bioinfodata.org/Blast4OneKP/species?id=800
Coleochaete scutata	Chlorophyta	VQBJ	https://www.bioinfodata.org/Blast4OneKP/species?id=759
Chondrus crispus	Rhodophyta	UGPM	https://www.bioinfodata.org/Blast4OneKP/species?id=1157
Gracilaria lemaneiformi	Rhodophyta	IKWM	https://www.bioinfodata.org/Blast4OneKP/species?id=1162

BACTERIA			
**Organism name**	**Family**	**Organism ID**	**website link**
Rhodopseudomonas palustris (strain ATCC BAA-98 / CGA009)	Bradyrhizobiaceae	258594	http://genome.microbedb.jp/cyanobase/Rhodopseudomonas
Anabaena variabilis ATCC 29413	Nostocaceae	240292	http://genome.microbedb.jp/cyanobase/AVA
Arthrospira platensis NIES-39	Arthrospira	696747	http://genome.microbedb.jp/cyanobase/NIES39
Microcystis aeruginosa NIES-843	Chroococcales	449447	http://genome.microbedb.jp/cyanobase/Microcystis
Prochlorococcus marinus SS120	Prochlorococcaceae	167539	http://genome.microbedb.jp/cyanobase/SS120
Nostoc punctiforme ATCC 29133	Nostocaceae	63737	http://genome.microbedb.jp/cyanobase/NPUN
Escherichia coli (strain K12 / DH10B)	Enterobacteriaceae	316385	http://www.uniprot.org/taxonomy/316385
Staphylococcus aureus	Staphylococcaceae	703339	http://www.ncbi.nlm.nih.gov/genome/?term=txid703339
