# Supplementary material for: Evolution of RLSB, a nuclear-encoded S1 domain RNA binding protein associated with post-transcriptional regulation of plastid-encoded rbcL mRNA in vascular plants
Source: BMC Evol Biol. 2016 Jun 29;16:141. doi: 10.1186/s12862-016-0713-1 (PMC4928308; doi:10.1186/s12862-016-0713-1)
Supplement: Additional file 3: Figure S2. — The primer sets used to amplify regions specific only for RLSB-a and for RLSB-b are shown. The amplified product sizes are 150 and 153 bp respectively. The regions where the left and right primers bind are indicated by arrows. (PDF 162 kb) [file 12862_2016_713_MOESM3_ESM.pdf]

Additional File 3: Figure S2

```
RLSB-a

   LEFT PRIMER        CCACTTCCATAACCCAGCAT
   RIGHT PRIMER       ATTTCACTCCAGGGGCACTA

   Product size 150bp

  1 ATGGTTCAAATTTCTGCATCATAGCTAAGCTGCTGACAACAGCGACAGAGACATATCTGG

 61 TACTTGTTGACCATGCAAAGGTGAACTGTCGCTAGAGAACCTGCCAACCTGTTGAAGTCC

121 AATACTGACCAGTGGTGGGTAGTTCTCCACTTCCATAACCCAGCATCAGTCATGGAGAAG
                              >>>>>>>>>>>>>>>>>>>>

181 TATGTTGGACAGGAACAGGCAACTTCATCCCGCAGACAAGATAGGGTACAAGGTTTCCAG

241 GCTGCAGAACATTGGTGTTTCGAATTGCCTCATTCCTAGTGCCCCTGGAGTGAAATGTTG
                                        <<<<<<<<<<<<<<<<<<<<

301 TGATATCATTTTATCTGTCCGGAGAATGTAGCTTCATTGCATTCATGTTATGTCAAAACT

361 AGTCGGGCTGCCCAAGCACTCCGTGCCAACAACTTGCATCTGCCCATGAAATAGATTCTG

421 AAATTTTAGCACCGT
```

```
RLSB-b

   LEFT PRIMER        ATCAACAGAAGAAGCGCTCG
   RIGHT PRIMER       TAACTAACCCCACGCTCACC

   Product size 153bp

  1 ATGCCGCCGCCCTCCCTCGTCTCCATCCTCCCCGCCGCGTCCATCTCGCCACGAAGACGC

 61 CAAGCAAAGCACCCCAAGACCCCCACCGCGAGCCATCCCTTCTTCTCCCGAAGACTTGTG

121 TCCCAACCGCCGCGCCACGCCTCGATCTCCCCCTCCGCTGCCGGCGGGAGCGGGAGCGAC

181 GAGCTCCACCTTCTCGAGAAGCCGTTACCTGCCGTCGCAGAGGATAACGAGGCCGAGGAG

241 GAGCTCGAGTCTGCTCCGGTGTCATCAACAGAAGAAGCGCTCGCGCCTTTTCTCAAGTTC
                           >>>>>>>>>>>>>>>>>>>>

301 TTCCAGGTGAAGAGCACCGACCCCGACGTGGGCGCAGACACCGCGGATGGCGAGGTGCCG

361 GAGGAGGCGTCAAGGAGAGATGTATTGTCCGCCGCTGGTGAGCGTGGGGTTAGTTA
                                        <<<<<<<<<<<<<<<<<<<<
```
