# Supplementary material for: Evolution of RLSB, a nuclear-encoded S1 domain RNA binding protein associated with post-transcriptional regulation of plastid-encoded rbcL mRNA in vascular plants
Source: BMC Evol Biol. 2016 Jun 29;16:141. doi: 10.1186/s12862-016-0713-1 (PMC4928308; doi:10.1186/s12862-016-0713-1)
Supplement: Additional file 4: Figure S3. — Multiple sequence alignment using T-coffee sequence aligner software (http://tcoffee.crg.cat/apps/tcoffee/index.html) of the Arabidopsis SDP S1 ribosomal protein and RLSB shows that these two proteins share only very little sequence similarity, identifying as distinct proteins. (PDF 533 kb) [file 12862_2016_713_MOESM4_ESM.pdf]

Additional File 4: Figure S3

```
SDP             MDVLALSSSASAAAPSASLAGKFLSFPSRVRVRRNRENLLAKQKKFLVSASKREEPKLNEWDQMELNFGRLLGEDPKLTL
RLSB            MQTLLCQPCKSLPILTASSSSSLIRSSGDVR-------------------------------------------------
                *:.*  ... * .  :** :..::  .. **

SDP             AKIVARKVDPEASFIDIEKSFYKNKGKIPEVEEIPLDWSKDNKKKSTSSLDGLKLVKPVLKDGVKFERPVMKKPSPVLKK
RLSB            --------------------------------------------------------------------------------

SDP             PLVEAVAAPKVQRLPNVILRKPSSFYTSNGDDEESKLRLKPNLTLKMRNERENERF-----------------SDMTLLR
RLSB            ECIDFRASEKVSKFQFHVTLSPFAFRGFS-ICREFAVRGAYGIR-----------FCSREDVSGVGNGGIVAEEEIELLN
                  ::  *: **.::   :  .* :*   .   .*  :*   .:            *                 .:: **.

SDP             KPEPVSVVAEEEDKPLSDDLTMEEGEQEGGTYSQYTLLEKPEARLQPVNVEEEVGDSGGVESSEIVNNSIQKPEARPELE
RLSB            KPNPLPKSENEESGKADD--------------------------------------------DAILEPFLKFFKPEEEGE
                **:*:.   :**.   .*                                            . *::  ::  :.. * *

SDP             NIEKEVADSGVLESSEIENNSIPTEMQLNSEMSSEEKT----INSDPLERIPSKPI------------------------
RLSB            GIESEVSDETDRVSVEYY-DPKPGDFVVGVVVSGNENKLDVNIGADMLGTMLTKEILPLYDKELDYLLCDLKYDAEEFLV
                .**.**:*.    * *   :. * :: :.  :*.:*:.    *.:* *  : :* *

SDP             ------------SQTIVEASLQGKPQRLDPSSAEPSVPNIGKPSVVNHEGRQVSVELKGPP---TRSSLEENDWNKAESL
RLSB            NGKMGIVKDDDEGVEIAEFARQGRP-----------VVEIGTVVFAEVLGRTLS----GRPLLSSRRYFRRIAWHRVRQI
                            .  *.* : **:*           * :**.  ..:  ** :*    * *   :*  :..  *::...:

SDP             VKTELRADVELISSSTRGFAVSYGSLIGFLPYRNLAAKWKFLAFESWLRRKGVDPSPYRQNLGVIGGQDVTSKSPSPDSS
RLSB            KQLNEPIEVKITEWNTGGLLTRIEGLRAFIPKQEL---------------------------------------------
                 : :   :*:: . .* *: .   .* .*:* ::*

SDP             LDSEVATTINGEVSSDMKLEDLLMVYDREKQKFLSSFVGQKIKVNVVMANRNSRKLIFSMRPRENEEEVEKKRTLMAKLR
RLSB            -------------------------------------VGRRFLVQITRLNEDKNDLILSEKVAW----------EKLYLR
                                                     **::: *::.  *.:...**:* :                 **

SDP             VGDVVKCCIKKITYFGIFCELE--GVPALVHQSEVSWDATLDPASYFKIGQIVEAKVHQLDFALERIFLSLKEITPDPLT
RLSB            EGTLLEGTVVKILPYGAQVKLGDSSRSGLLHISNITRR---------RIGSVS--DVLQVDESVK--VLVVKSLFPDKIS
                 * :::  : **  :*   :*   . ..*:* *:::           :**.:   .* *:* :::  .* :*.: ** ::

SDP             EALESVVGGDNDQLGGRLQAAELDAEWPDVESLIKELEMVEGIQSVSKSRFFLSPGLAPTFQVYMAPMFENQYKLLARAG
RLSB            LSIADLESE-----PG-LFISDREKVFTEAEEMAKKY--REKMPLVATSPISDRPPITSSFPQ-----------------
                 :: .: .       * *  :: :  :.:.*.: *:    * :  *:.* :   * ::.:*

SDP             NRVQELIVEASLSKEEMKSTIMSCTNRVE
RLSB            GKDEEIYANWEWFKFESQ-----------
                .: :*: .: .  * * :
```
